# Disruption and recovery of family planning, contraception and other sexual and reproductive health services in Brazil with COVID-19 pandemic: a mixed methods approach

**DOI:** 10.1186/s12978-025-02088-w

**Published:** 2025-08-08

**Authors:** Luis Bahamondes, Jose G. Cecatti, Aline Munezero, Rachel E. Soeiro, Karayna G. Fernandes, Samira M. Haddad, Silvana Ferreira Bento, Karla S. Padua, Vilma Zotareli, Charles M. Charles, Montas Laporte, Caron Kim, Vanessa Brizuela, Moazzam Ali, for the WHO HRP Social Science Research Team, Mariana B. Rogerio, Eunice Chomi, Seni Kouanda, Flore Marie Gisèle Donessouné, Armel Sogo, Kun Tang, Yueping Guo, Hanxiyue Zhang, Yifan Zhu, Ge Yang, Chunxiao Peng, Xizhuo Xie, Hao Wang, Deda Ogum Alangea, Kwasi Torpey, Emefa Judith Modey, Adom Manu, Ernest T. Maya, Rozina Karmaliani, Laila Ladak, Arusa Lakhani, Marina Baig, Yasmin Parpio, Salima Somani, Pisake Lumbiganon, Jen Sothornwit, Nampet Jampathong, Somporn Rungreangkulkij, Marleen Temmerman, Ferdinand Okwaro, Abdu Mohiddin, Massimo Mirandola, Maddalena Cordioli, Alessia Savoldi, Simone Garzon, Stefano Uccella, Nigel Sherriff, Alexandra Sawyer, Catherine Aicken, Jörg W. Huber, Jaime Vera, Deborah Williams, Hamsadvani Kuganantham, Grace Kapustianyk, Igor Toskin, Soe Soe Thwin, Armando Seuc, Gabriela Garcia Camacho

**Affiliations:** 1 Department of Obstetrics and Gynaecology, School of Medical Sciences, the University of Campinas, and Cemicamp, Campinas, SP Brazil; 2 Department of Obstetrics and Gynaecology, Jundiai School of Medicine, Jundiaí, SP Brazil; 3 School of Medicine, University Unoeste, Guarujá, SP Brazil; 4 Department of Sexual and Reproductive Health, World Health Organization, Geneve, Switzerland

**Keywords:** COVID-19 pandemic, Contraception, Family planning, Mixed methods

## Abstract

**Background:**

Limited scientific evidence is available on the impact of COVID-19 on Sexual and Reproductive Health (SRH). Some data shows that severe disruptions in SRH services expose women to preventable health risks. This study has the objective to provide a better understanding of the impact of COVID-19 on family planning and contraception among other SRH services and strengthen policies and services to be more responsive to community needs.

**Methods:**

A mixed quantitative and qualitative methods were used to assess SRH service availability and readiness, and clients’ and providers’ perceptions in some Brazilian COVID-19-affected areas. Interviews were performed at baseline and 6–9 months later. It was conducted in three cities from the São Paulo state, Campinas, Jundiai, and Santos. The health facility level involved five questionnaires for assessment of infrastructure availability and readiness to provide SRH services and a qualitative survey to elicit health services providers´ perspectives. The qualitative in-depth interviews (IDI) with the participants and Focus Group Discussion (FGD) with women and partners were conducted using a semi-structured interview, recorded and transcribed. A thematic content analysis was then performed.

**Results:**

The settings studied had different characteristics of geographic size, population, and organisation of health services that influenced the way they faced COVID-19. During the baseline interview, the population mentioned fear of seeking health support in the PHU; however, this situation was still observed in Santos in the end-line interview. Antenatal and post-partum care was offered to the population; however, other demands, such as contraception were not. In the interviews, contraception services were considered a priority. The different speeches and behaviours of the government leaders caused discredit to the health measures recommended by the WHO. There is a need for alignment among health managers at municipal, state, and federal levels.

**Conclusions:**

The municipal administration played a key role in the fight against COVID-19 together with the coordinators of the health services. Contraception was not considered a priority during the pandemic. However, participants reported they should be considered essential services in future pandemics. The PHU readiness offered antenatal and post-partum care, while other population needs were not prioritized.

## Abstract (plain language summary)

The objectives of this study were to assess the impact of COVID-19 on family planning and contraception and to strengthen policies and services to be more responsive to community needs in Brazil. We used mixed quantitative and qualitative methods to assess SRH service availability and readiness, and clients’ and providers’ perceptions in three COVID-19-affected areas. Interviews were performed at baseline and 6–9 months later. We used five questionnaires for assessing infrastructure availability and readiness to provide SRH services and a qualitative survey to capture health service providers´ perspectives. The qualitative in-depth interviews (IDI) and Focus Group Discussions (FGD) with women and partners were conducted using a semi-structured interview. A thematic content analysis was then performed. The settings had different characteristics influencing the way they faced COVID-19. During the baseline interview, the population mentioned fear of seeking health support in the PHU; however, the same was still observed in one city in the end-line. Antenatal and post-partum care was offered to the population; however, contraception that was considered a priority was not. Contradictory speeches of the government leaders caused discredit to the health measures recommended by the WHO, evidencing the need for alignment among health managers at all levels. The municipal administration plus the coordinators of the health services played a key role in the fight against COVID-19. Contraception was not considered a priority during the pandemic, although participants said they should be essential in future pandemics. The PHU readiness offered antenatal and post-partum care, while other population needs were not prioritized.

## Background

In December 2019 there was the announcement of a new disease called COVID-19 that was first reported in China and then rapidly spread globally, being characterized as a pandemic by the World Health Organisation (WHO) just three months later in March 2020 [1]. As of 16th February 2024, there were over 774 million confirmed cases and 7 million deaths reported globally [2]. This pandemic was soon associated with a significant rise in demand on health systems for the care of people with COVID-19 infection, demanding a huge amount of financial resources to be invested, for supporting medical care, as the only immediate way to face the new condition at that moment. This unprecedented increased demand together with the recommended restrictions on the mobility of people to avoid higher dissemination of the virus were suspected to lead to disruptions in the provision of essential services, including sexual and reproductive health (SRH) services [2–5].

Previous experiences from other outbreaks suggested that some services could be not available because the institutional and human health resources were redirected for COVID-19, or even because there are beliefs that healthy people should avoid going to health facilities if not sick [3, 4]. Maintaining the continuity of essential health services while keeping people safe during the response to disease outbreaks is essential to the prevention of both direct and indirect mortality [6, 7].

Although limited and controversial scientific evidence is available on the role of COVID-19 on SRH, there are reports on important disruptions in those services that put women at high risk of preventable conditions [5, 7, 8]. With the extra amount of work due to COVID-19 cases, possibly healthcare providers may not have enough time or motivation necessary to guide contraceptive methods [9]. It seems that this limited availability of contraceptive methods represented a significant number of additional unplanned pregnancies, especially in low-resourced settings [6]. Several studies have hypothesized the potential impacts of the COVID-19 pandemic, even using complex mathematical models, with different levels of reduction in essential SRH services, on estimates of alarming numbers of unplanned pregnancies, unsafe abortions, gender-based violence (GBV) and maternal deaths [5–7, 9, 10].

Our study evaluated the impact of COVID-19 on the health systems'capacity to provide SRH services. The data provided a better understanding of the impact of COVID-19 on these services at the subnational and facility level, which will help policymakers and health managers develop and strengthen policies and services to be more responsive to community needs. In brief, the research was expected to identify the key gaps and barriers to the delivery of important aspects of comprehensive SRH services in health emergencies. It would also provide a set of strategies, which can be applied in any setting, to assess health facility and system readiness to respond to health emergencies in the future.

### Country context: health, health care, and disease surveillance in Brazil

The SARS-CoV-2 (COVID-19) infection affected the world as one of the largest pandemics in history. The WHO estimated more than 7 million deaths worldwide due to COVID-19, with almost 700,000 deaths in Brazil [11]. In March 2024, more than 500 million doses of COVID-19 vaccines were already administered in Brazil [12].

### Healthcare system and context for Brazil

In Brazil, 74% of the population depends on the national health service (SUS-Sistema Único de Saúde) as the main source of medical care, including the provision of free contraception: combined oral contraceptives (COCs), progestin-only pills (POPs), levonorgestrel (LNG) as emergency contraception (EC) pills, once-a-month injectable and 3 months depot medroxyprogesterone acetate (DMPA) injectable, copper intrauterine devices (Cu-IUDs) and condoms. The etonogestrel (ENG) implant and the levonorgestrel-releasing intrauterine systems (LNG-IUSs) are not available in the public sector, except at some teaching hospitals. There are important bureaucratic barriers to accessing contraceptive methods, especially among those on a low income [13]. The COVID-19 pandemic has severely disrupted the healthcare system nationwide and the fragility of the public health system has become more evident. The pandemic posed a challenge for many women with SRH needs: some services have been closed because many health authorities considered them non-essential, and among healthcare professionals, there has been a high number of deaths, many have been redeployed to care for the COVID-19 cases, and others have become exhausted and left the profession [14].

All these situations have negatively affected the health care system, including SRH services and the deleterious effects were more evident among low-income people. Although equitable and safe access to contraception was declared a human right five decades ago, family planning (68%) and antenatal care (56%) services have been the most frequently disrupted during the pandemic, undermining women’s rights and the role of SRH services [13].

The objectives of our study were to explore the status of, availability, and health service readiness to provide SRH care during the COVID-19 pandemic and after recovery in Brazil, focusing on the selected SRH services: contraceptive provision and family planning. Specific objectives were to assess the availability and health facility readiness to provide these services; to qualitatively assess the risk perceptions, stigma, barriers to access, utilisation, and quality of the services, from clients’ and healthcare professionals’ perspectives; and to assess the post-pandemic recovery of the facilities providing these services. Our current manuscript presents mainly findings related to contraception, and family planning as SRH services.

## Methods

### Study context

The minimum criteria for the selection of health facilities within the selected geographic sites were outlined including the availability of human resources i.e., SRH service provision primarily on their qualification to provide services in contraception, family planning and other SRH and support for women during pregnancy. In addition, the diversity of hospital capacity, urban/peri-urban settings, being a COVID-19 treatment centre and the willingness of the providers in charge to participate in the study, and how the COVID-19 response has been organised in terms of treatment centres. In Brazil, the following sites were chosen: Campinas, with a teaching tertiary hospital, and Santos and Jundiai, with similar population characteristics, tertiary and primary health services provided by the municipality.

#### Selection of participants

##### Healthcare providers (HCPs)

The HCPs were purposively selected to participate in the in-depth interviews (IDIs) and the health facility assessment in each service in the different cities. Three HCPs per service were invited to participate in the IDIs, and only those 1) who delivered SRH services, 2) were most knowledgeable about readiness and availability of SRH services, 3) were stationed in the SRH clinic/hospital and had been working at the clinic/hospital for at least 6 months before the pandemic started were selected for inclusion in the study. Participation was voluntary and the HCPs were selected after obtaining information from their facility.

##### Clients

The study invited women of reproductive age (18–49 years) who were in the waiting room at the services, who were seeking contraception, abortion, post-abortion care, STI care and treatment, or GBV care and support services in the selected facilities.

##### Partners

The study invited men who were at the same service with their wives/partners. They were in the waiting room at the services in the same cities and accomplished the study criteria.

### Qualitative methods in health systems study

#### Study design

We used the qualitative method to assess clients’ and providers’ perceptions of the availability and readiness of these services. The study was carried out on two levels, baseline and end-line. In both moments, the women, partners and HCP participated in IDI. Women and men also participated in the FGD.

#### Study settings and timing

The study in Brazil was carried out in three cities from the three metropolitan regions of the State of São Paulo, Campinas, Jundiai, and Santos, located in the Southeast region of Brazil (Table 1). Baseline and end-line data in Brazil were collected from October 2021 to June 2022 in Campinas, from February 2022 to November 2022 in Santos, and from April 2022 to December 2022 in Jundiai.

**Table 1:** Settings for SRHS and target sample sizes for IDI and FGD

Location	Facility and setting	Time	HCPs	Clients	Partners	Total
Campinas	Caism – Woman´s Hospital Tertiary level facility	Baseline	3	27	19	49
		End-line	3	24	12	39
Jundiai	HU – University Hospital and Health Center (Tamoio) – Tertiary and primary level	Baseline	2	11	9	22
		End-line	1	11	10	22
Santos	Health Center (Santos) Primary health unit	Baseline	3	9	10	22
		End-line	3	5	9	17
Total		Baseline	8	47	38	93
		End-line	7	40	31	78
Total General			15	87	69	171

#### Sampling, recruitment

Purposive sampling was used with target sample sizes described in Table 1, which show the distribution of the number of interviews by city, type of interviewee, and time of interview. Following the WHO protocol for all participating countries, each sample for the qualitative component would be selected based on specific criteria, as well as specific country contextual factors. For the IDIs, a sample of 10–15 women (or until saturation) and 6–12 partners (or until saturation) would be recruited. In addition, approximately one to two healthcare providers per health facility should be purposively selected. For the quantitative component, it was estimated that up to 20 women and partners per site would be sufficient, while one HCP would be selected by health facility [15]. All the invitations and interviews were done personally in the baseline when they were performed in the services in a specific place. During the end-line, there were 27 interviews (11 from Campinas and 16 from Jundiai) done by phone due to the restricted time and availability of the participants.

#### Health Care Providers (HCP)

In Jundiai, at the baseline interview, only two HCPs agreed to participate in the study. In the end-line, there was a refusal to make the second interview. So, in Jundiai, there were only two professionals at baseline and only one at the end-line.

#### Clients

The study initially recruited a purposive sample of a total of 98 women (53 at baseline and 45 at end line); but only 87 of them were of reproductive age seeking contraception, abortion or post-abortion care, STI care and treatment, or GBV care and support services.

#### Partners

The study initially recruited a purposive sample of 73 men (40 at baseline and 33 at end-line) but only 69 were partners who accompanied women to the hospitals for individual interviews.

#### Data collection and data management

The qualitative interviews: firstly, Participant Information Sheets (PIS) were filled out with participant’s data. Then, IDI with the participants and organized FGD with women and partners were all recorded. The interviews were conducted using a semi-structured guide and the same occurred for FGD. All interviews were recorded into two digital portable recorders (Sony®, model: ICD-PX240) and stored directly in an electronic file into a cloud “Drive” from Google. Subsequently, the interviews were transcribed and reviewed by research personnel who conducted the interviews.

In Campinas, the interviews and FGD were organized in specific rooms. The FGD with the women´s community was carried out at a non-profit organisation. In Jundiai, the interviews and FGD were organized at a teaching hospital and one Primary Health Unit (PHU). In Santos, the interviews and FGD were organised at the PHU. The women´s FGDs were carried out with two experienced female researchers, while the men´s FGDs were carried out with two experienced male researchers.

#### Analysis

For data analysis, specific guidance was followed [16, 17]. First, the transcriptions were read, and meaningful units were marked in the speech of participants. Categories for analysis were created from the meaningful units. These categories were composed of codes applied to portions of the text and later similar passages were grouped by category in all interviews. Afterwards, a thematic content analysis of each group of texts was performed, based on proposed categories of analysis and study aims. The qualitative data analysis was performed according to the city, considering that in each city there is a different health organisation. We considered the opinion of women, partners and health authorities, comparing baseline and end-line with the FGDs from women and partners.

The analyses were done comparing the participants (clients, partners, and health care providers) at baseline and end-line in the three cities. A team of experienced researchers carried out the analysis. Afterwards, the analysis was presented to a group of researchers for data validation. Some speeches from participants are reported as examples to justify the thematic results in the order they are presented. Additional detailed information on the methods of the study and its analytical approach is available in a previous publication focusing on the research proposal which was implemented also in some other countries [15]. In this article, we are mainly focusing on data regarding perception of and access to contraception among SRH services.

### Quantitative methods

#### Design

This study also adopted a quantitative approach to assess health facilities’ SRH service availability and readiness, and clients’ and providers’ perceptions of the availability and readiness of these services in some Brazilian COVID-19-affected areas. The study was carried out using a structured questionnaire called “General questionnaire” for all participants.

The individual level involved clients (and their partners) and health services for data collection. The health facility level involved a quantitative assessment of infrastructure availability and readiness to provide SRH services and a qualitative survey to elicit HCP perspectives on the same. There were two data collection points, baseline and end line, where data was collected in an 8 to 9-month interval. The general questionnaire and the questionnaires about the facility infrastructure were filled out in baseline and end line.

#### Data collection and data management

HCP, women and partners were identified and invited to participate similarly to what was followed for the qualitative component.

The interviews of the 5 participating HCP/health authorities were scheduled according to their convenience. Some interviews were by telephone at baseline and end line. The participants filled out the following questionnaires in baseline and end line: (1) WHO Service Availability and Readiness Assessment (SARA) guide; (2) WHO Health Emergency Facility Assessment Checklist in Emergencies; (3) WHO safe abortion assessment tool; (4) WHO GBV health system readiness tool; and (5) WHO STI assessment tool.

#### Analysis

The analysis was done by comparing T1 (baseline) and T2 (end line) in each city (Campinas, Jundiai and Santos) for 156 clients and 5 HCP/health authorities, performed by the statistician team from the WHO.

### Ethical aspects

The Ethical Committee of the University of Campinas approved the protocol, which was also approved by the WHO Ethics Review Committee and the Brazilian National Council on Research Ethics. Each participant signed an informed consent form before starting the interview after clarifying all doubts. For the interviews done by phone, a recorded verbal consent was obtained and a photo from the term signed was also sent.

The participant was informed that the data collected was held in strict confidence and that only numbers and letters identified the information collected. The identifiable information was stored in a separate encrypted hard drive and was linked to the data through an identifier. Access to identifiable information is restrained with the highest scrutiny. Due to challenges in the level of COVID-19 transmission, the study protocol was flexible to adjust to these challenges, for instance, if face-to-face was not feasible, then remote options such as online, could be employed. The main intention was to minimize the risk of COVID-19 transmission to both participants and interviewers.

There were no financial incentives provided to participate in this study. However, the participants and their companions received compensation to cover incidental costs of their participation such as the use of telephone, travel expenses, etc.

The audio tapes did not have individual identifiers such as name and address to ensure the privacy and confidentiality of the participants. The audio tapes were destroyed after the data had been transcribed and reviewed by the research team.

## Results

### Findings of the qualitative component

As outlined in Table 1, 87 clients, 69 partners, and 15 HCPs (baseline and end-line) were interviewed. Almost all the participants (95.3%) live in urban areas with water and sewage, 36.2% were over 40 years old, 43.8% self-declared as white and 40.3% as brown. Their family incomes were predominantly in the lower ranges of one to two minimum salaries in Brazil (US$ 400.00). Around 38% of them were employed with a signed contract and 27.5% were self-employed, 38% had completed high school. About the religion, 41% declared to be catholic and 31% evangelical. Most participants declared to be married (40.3%) and 43.8% declared living together/single with a partner (Table 2).

**Table 2:** Socioeconomic characteristics of participants (women, men and healthcare providers)

Characteristics	n	%
Age		
18–25	33	19,30
26–35	57	33,33
36–40	19	11,11
> 40	62	36,26
Housing		
Urban Areas with water and sewage	163	95,32
Urban Areas without water and sewage	3	1,75
Rural areas with water and sewage	3	1,75
Rural areas without water and sewage	2	1,17
Skin colour		
White	75	43,86
Black	25	14,62
Brown	69	40,35
Yellow	2	1,17
Indigenous	0	0,00
Family income		
Until one minimum salary (US$ 200.00)	41	23,98
US$ 201 – US$ 565	72	42,11
US$ 566 – US$ 945,00	33	19,30
US$ 946 to US$ 1320	12	7,02
< 1320,00	13	7,60
Occupation		
Employee with a signed contract	65	38,01
Unpaid	46	26,90
Employee without a signed work permit	13	7,60
Self-employed	47	27,49
Education level		
Elementary School completed	16	9,36
Elementary School Incomplete	11	6,43
High School completed	65	38,01
High School Incomplete	27	15,79
College incomplete	19	11,11
College completed	20	11,70
Post-graduation	13	7,60
Religion		
Catholic	70	40,94
Evangelical	53	30,99
Jehovah's Witness	4	2,34
Spiritist	8	4,68
Protestant	4	2,34
None/No Informed	24	14,04
Believes in God	2	1,17
African roots (Umbanda, Candomble)	6	3,51
Marital status		
Married	69	40,35
Living together with a partner	37	21,64
Single with a partner	38	22,22
Single without a partner	17	9,94
Widow/Widower	1	0,58
Divorced	9	5,26
TOTAL	171	100,00

### HCP’s perspectives and experiences

#### Service delivery

SRH services were maintained in all three cities. However, some specific characteristics were observed in each service during the management of the pandemic. In Campinas, the hospital that participated in the study was a tertiary referral hospital in women's health. The measures taken were carried out in such a way as to cover each case since the arrival of the women at the emergency room that contained information, separation of the spaces for those with flu symptoms and those without and the spaces for care, hospitalisation, and reception of the baby in case of delivery. This was despite having received the mobilisation of resources only after a certain time after the beginning of the pandemic. As the hospital was well structured, both from the point of view of professionals and resources, the measures taken were well developed. In Jundiai, the organisation of the response to COVID-19 was done in a way to cover all the health sectors of the city with a flowchart of how the service should be, which health authorities and patients knew. There was a website that the population could access for all the information about the disease, also how to behave themselves in case of eventual symptoms. This provided security to the population and healthcare professionals and the correct referral of care. The healthcare professionals received information, and this facilitated the performance of their work. In Santos, on the other hand, there were changes in the government's recommendations regarding the confrontation of the pandemic and the population felt lost about where and to what service to seek in case of symptoms of COVID-19, whether in the PHU or the hospital. One of the health authorities mentioned that the city did not prioritize SRH; all actions were focused on the healthcare of the infected population.

*"What I noticed is that they were also afraid to come to the PHU, so many I think dropped out a little bit and then they picked up again. That's I noticed. Demand decreased, I think because they were afraid to come to the unit*"[Authority Maternity SH02FC, Baseline]

#### Workforce (human resources)

In all cities, human resources were affected by COVID-19. The HCPs who were over 60 years old or had any comorbidity were turned away. Those who kept taking care of the population, many times were infected with COVID-19 and took sick leave for 15 days at least. It made the head of the services change the routine and the focus was directed mainly to antenatal care and post-natal care. In Jundiai, at the end-line, it was reported that the hospital used the resources received by the Federal Government and hired some doctors and the payment of overtime for these professionals and nurses during the pandemic, as well as for the purchase of COVID-19 tests and personal protective equipment (PPE).

The authorities reported that they felt stressed about COVID-19, but the intensity of this feeling was different. The reasons the authorities said they felt stressed were because of work-related issues such as fear of infection and death, and work overload because several employees were off work.

#### Information systems

Jundiai used technology to pass information to the population and healthcare professionals had lower complaint rates than those that randomly passed information to the population through social media. Campinas and Santos used the telephone and WhatsApp from the service to get the population informed. In Campinas, telemedicine worked well but the same was not observed in Santos, because the services did not have good internet access and did not have adequate equipment.

"... *we developed the orientation for patients of the network and this service is maintained until today, which is called tele orientation. We provide triage support and help in the management of patients from the entire region, which we are a reference, including 40 cities in the region. We have a call centre every day, from 11AM to noon, in which doctors from these locations contact us to discuss the cases and then we select those that really need to be referred*..."[Authority 3]

The authorities reported that the information received was scientifically based and was considered adequate. However, much of the information given by the media was erroneous.

#### Access to essential medicines and supplies

The cities informed that generally there was not a lack of essential medications and supplies. Santos mentioned at baseline that there were difficulties in the supply of some medications that were restricted because the production in the pharmaceutical industry suffered an impact due to COVID-19. In the end-line, it was solved and no problem with supplies was mentioned. Campinas mentioned that the prices of some supplies were higher, for example, gloves, and alcohol, then they negotiated the quantity and delivery time, and the hospital did not suffer any problem with supplies of essential products.

#### Financing

All cities received financial sources from the Federal Government. Campinas received in the end-line, so all actions in baseline were done with its own resources. In Santos and Jundiai, the funds received were addressed to the hospitals to adequate their structure to take care of the severe COVID-19 events. The funds were not addressed to the PHU.

#### Demand for, availability and delivery of contraception services during COVID-19

The services, in the three cities, were structured to offer antenatal care and post-natal care that were prioritised. Contraception services were not prioritised. The family planning services in the Jundiai and Santos were affected due to the lack of gynaecologists for routine examinations, drug prescriptions and routine exams. In Campinas, the family planning services were kept, but the population, in the three cities, was afraid to seek the service and get infected with COVID-19. The public transportation was reduced, and it was crowded. Santos and Jundiai cities observed the same behaviour of the population afraid to seek health services.

### Clients’ (and partners’) perspectives and experiences

#### Knowledge of COVID-19, risk and prevention

In both rounds of the survey the participants, men and women, knew how transmission occurs, the symptoms, and where to seek medical attention in case of suspicion of having contracted COVID-19. In their discourse, it was mentioned that at the beginning of the pandemic, there was not much knowledge about the disease, information was scarce and distorted, and not even healthcare professionals knew about the disease. In addition, many fights between the rulers were more concerned with politics than with the health of the population. Because of this, there was a lack of trust in the information received and the dissemination of a lot of false news, which caused people not to follow the guidelines because they didn't know what to do when faced with so much contradictory information. There was a lack of seriousness and transparency from the authorities.

#### Leadership and governance

In Brazil, there was a serious political problem. The Federal Government was not aligned with the São Paulo State governor (where the study was performed). Thus, there were many public arguments about the procedures which should be adopted and followed.

*"...They [authorities] talking on television to wear a mask, then they [authorities] appear having a family party, crowding together"[Woman, 1004-1016, baseline]*

*"There are politicians who influence totally the opposite, influence not to wear a mask, there is no danger, the president of the Republic himself did that several times"[Woman, 1004-1004, baseline]*

In both rounds, all participants obtained information about COVID-19 through health services, healthcare workers, social media, social networks, and pamphlets. It was mentioned that reliable information was found on government websites (Ministry of Health, WHO), and reputable TV news. One resource used to confirm the veracity of the information received was to check these with other communication sources. After this first moment, there was an appeasement among the rulers who started to think more about the population, the information started to be standardised, thus passing more adequate orientations to the population. From the report of all participants, from the end-line, it was observed that criticism of the media and the government's actions were softened, probably due to the action of time to the occurrence of the pandemic.

#### Contraceptive method used, changes to contraceptive use, and pregnancy intention

In Jundiai, most women reported that during the pandemic they needed a prescription to obtain a contraceptive and a doctor's appointment for a new choice. It was a barrier because it was difficult to schedule an appointment and when they finally got it, the doctor was on sick leave because was infected by COVID-19. The same situation was not experienced by the partners who did not need to change methods during the pandemic period**.** In Campinas, some participants reported that their partners had used the SRH services to change contraceptive methods and had not encountered any difficulties in scheduling an appointment. This is because the service at the tertiary hospital was not closed. However, in the end-line, it was mentioned that not all public services [PHU] have contraceptive methods available. In those that did, it was quick to make an appointment, but if an exam, such as an ultrasound, was needed, the waiting time was long to get it. Consensus existed in the interviews and FGD of women, they reported difficulty in scheduling a doctor's appointment. One woman in the baseline interview reported that she needed to have a consultation to check the IUD and it took more than a year trying, and she was only able to have the revision done in another service at a tertiary hospital**.** In Santos, all the participants, interviews and FGDs women and partners, baseline and end-line, mentioned that they did not seek the service for contraceptive methods and neither did their partners. They continued with the methods they were using, mostly the pill and condoms. Some of them decided to change methods due to some discomfort with them and made this contraceptive change on their own, for example, they exchanged contraceptive pills for condoms.

#### Needs related to contraception

In Brazil, the reference to get information and contraceptive methods, for the general population, is the PHU. It is organised by the Government and it is free of charge. However, the barriers identified were: In Campinas, at the beginning of the pandemic, the PHUs were only accepting suspected COVID-19 patients and only hospitals were opened. Later, this was changed. The impact has rescheduled the appointments. Normally, there is a long waiting line for appointments and examinations, and then it gets worse. Jundiai did not close the PHU, and the population should go to the hospital with the indication of a PHU. Santos did not close the PHU, but the population did not know where to go, if in the hospital or a PHU. In Santos, there was a failure in communication with the population. In all cities, the number of healthcare professionals was reduced due to their age and comorbidities they had, so they stayed at home when it was possible, performing administrative tasks in home-office. Also, several professionals who stayed working got infected and got sick leave for at least 15 days or died.

#### ‘Patient journey’ – the process of seeking and receiving care

All women and partners show knowledge of how to get contraceptive methods and medical appointments, seeking the nearest PHU. The barrier was the long-time waiting in line for appointments and exams. This was told during the COVID-19 pandemic, to get worse, but it is always a long waiting line.

*"It is already difficult. During the pandemic it got harder*"(Woman 1004-3035, end line).

#### Perception of risk of (or safety from) COVID-19 infection during care-seeking

Many people stopped seeking medical assistance, in general, because they were afraid of becoming infected by COVID-19.

"*I will say you are more at risk on public transportation than in the hospital. In the hospital you have distancing, in public transportation you do not"* [Woman 1004-1049]

#### Barriers and facilitators to receiving care

One of the main barriers mentioned by the participants was the lack of doctors in the PHU.

*"And?: the doctor is not attending, he took sick leave because of the pandemic"[FG Women - Baseline]*

*"And?: I am almost 2 years waiting [consultation with gynaecologist]"[FG women - Baseline]*

It was said that the women who needed a visit with the gynaecologist for antenatal care had no problem making the appointment and doing the exams. However, this was not the reality for women experiencing other needs in other SHR services.

#### Psychosocial impact of the pandemic

The pandemic affected all health authorities, women and men. Women were more affected than men. The women´s worries were with being infected, dying, and leaving the family, while men´s were regarding their jobs and being unemployed.

#### Findings of the quantitative component

For the 156 clients and partners who participated in the quantitative component of the study in Brazil, Table 3 shows their sociodemographic characteristics and exposure to COVID-19. Participants'mean age was 34.6 years and 11.5 years of schooling, 55.8% were female, 64.3% currently cohabiting with a partner, 49.7% declared to be Hispanic, 50.6% were tested for COVID-19. Among them, almost 52% were positive for results of any test**.** Among these 87 women, 11 (12.8%) were pregnant at the moment of the interview and only 6 of them were at the facility looking for antenatal care. Regarding obtaining contraception, for those 129 individuals (82.7%) looking for this, 44 (34.1%) had access to family planning services, 25 (19.4%) had counselling only and 19 (14.7%) also got a method or prescription. The most prescribed or provided methods were levonorgestrel-IUD, COC and copper-IUD (data not shown).

**Table 3:** Sociodemographic characteristics of clients and partners and exposure to COVID-19 (*N* = 156)

Characteristics	n (%)	Mean
Age^a^ (range 16–64)		34.6 (155)
Gender		
• Female	87 (55.8)	
• Male	69 (44.2)	
Schooling (years)^b^ (range 10.9–12.1)		11.5
Cohabitation^c^		
• Without a partner	53 (34.4)	
• With a partner	99 (64.3)	
Ethnicity		
• Caucasian	37 (24.8)	
• Black	19 (12.9)	
• Asian	2 (1.4)	
• Hispanic	74 (49.7)	
• Other	7 (11.4)	
Are you currently smoking tobacco/living with someone who smokes? ^c^	40 (25.9)	
Have you been tested for COVID-19?	79 (50.6)	
Months since the last COVID-19 test (range 8.7–12.8)		10.7
Positive result		
• PCR	34 (43.0)	
• IgG/IgM	5 (6.3)	
• Antigenic	2 (2.5)	
TOTAL	156	

^a^Missing 1

^b^Missing 11

^c^Missing 4

#### Family planning services and referral

National family planning guidelines are present in the participating institutions according to the majority of participating HCP/authorities, equally at baseline and end-line. Similarly, the majority of them mentioned that there is an FP checklist and/or support materials available at the institution. Concerning the need for referrals to other FP services, only one of the HCPs said that there was such a need, at baseline and end-line. The reasons for these referrals in the past 6 months were contraceptive services not provided at the current location; adverse effects of the contraceptive method; and other reasons (data not shown).

#### Facility infrastructure for family planning

The questions on the infrastructure were answered by four HCPs both at baseline and end-line. For two of the items, we found a change over time in the professionals'response: the opening hours being convenient for patients, especially women and girls in the key population, including adolescents, and not being convenient opening hours by one of the professionals, but which at end-line was mentioned by all respondents as opening hours being convenient. The existence of the institution of reception to help, inform and guide patients was referred also to by the majority of respondents. The infrastructure items that were referred to as not existing in the FP service by more than half of the HCP were: the existence of separate waiting rooms, especially for adolescents and the existence of posters about violence against women and/or available brochures with no change over time. The existence of counselling rooms and examination rooms with sound isolation/protection so that others cannot hear and listen were reported as existing by half of the professionals equally at baseline and end-line (data not shown).

All participants, both at baseline and end-line, reported having signs in the clinic about the days and times when services were available; having a separate waiting room for adults for family planning/contraception services; having waiting areas and weather-protected seating; there is written information and materials available on the various contraceptive methods so that users can take the materials home to read.

#### Products/contraceptives

The information given by more than half of the HCPs is that their institution continues to stock contraceptive products locally, at baseline and end-line. The methods that all participants reported having in stock were: COC, progestin-only pill, progestin-only injectable contraceptive; copper-IUD and female sterilisation at request. However, diaphragm was not available in all facilities. The male condom, the female condom and the emergency contraceptive pill were the methods referred to by all professionals as having stock in their institutions at baseline, and there was a change in the end-line, where one of the professionals reported not having them in stock (data not shown).

The once-a-month injectable, implant and other methods that had been referred to, at baseline, as not being in stock by respectively 2, 3 and 3 of the professionals, decrease at end-line to 1, 2 and 2 of the professionals and contrary to that the number of professionals who said that there is a stock of this method over time increases from 2, 1 and 1 at baseline, to 3, 2 and 2 at end-line.

#### Human resources

Regarding training in contraception reported by healthcare providers, only one reported receiving training whereas two reported no training, the same was verified in the end-line, and there were no changes concerning this item. As for which professionals in the institution are involved in family planning care, specialists in gynaecology/obstetrics, general practitioners, nurses, obstetric nurses, and other professionals were mentioned by four of the professionals at baseline, and this number decreased to three professionals at the end-line.

#### Policies and plans

Of the five HCPs interviewed, four said that Brazil had defined a package of essential health services for SRH before COVID-19 and at the second round, end-line, this information was maintained by three professionals. According to the majority of the interviewees, in both periods there was identification of a set of essential health services to be maintained during the COVID-19 pandemic in Brazil. Regarding financial resources, all providers/clinics reported, at both time points, that additional government funds were available to ensure essential health services.

Concerning the dissemination and use of WHO and other technical guidance documents in the response to COVID-19 and the continuity of essential SRH services, all providers reported that they had received the latest guidelines/protocols; that the latest guidelines/protocols were shared with HCPs; and that they were being used to guide routine service delivery.

#### Maintenance of essentials health services

During the pandemic, the government policy for the care of essential services, according to more than half of the HCPs, was maintained as normal for emergency services and the other services were limited in terms of hours or who should receive the care, this in both moments of the study. Regarding the interruption of services provided by the institutions due to COVID-19, what was observed is that almost all the services provided were not interrupted, according to more than half of the professionals, both at baseline and end-line. One health professional reported that services completely disrupted due to COVID-19 were 24-h emergency/health units; blood transfusion emergency services; inpatient and critical care services; and emergency surgery (including obstetrics); this was noted both at baseline and at end-line.

The main causes of these changes/disruptions and/or changes in service utilization, given by the majority of HCPs at baseline, were: closure of outpatient services in accordance with government rules; closure of outpatient clinics for specific clinical consultations; decrease in outpatient volume due to no-shown of the patient; decrease in inpatient volume due to cancellation of elective care; insufficient staff to provide services; related clinical staff relocated to provide COVID-19 coping; changes to not treatment policies for care-seeking behaviour for fever symptoms (e.g., stay-at-home policies); restricted movement of government or public transportation making it difficult for patients to access health centres; financial hardship during the pandemic/social isolation. In the end-line, it was observed a decrease in the number of HCPs who reported as one of the main reasons for insufficient staff to provide services.

Some causes that were first observed in the end-line were: the closure of population screening programs for cervical cancer, mentioned by two professionals in the baseline and by three in the end-line; and insufficient PPE for healthcare professionals, which had not been mentioned by any professional in the baseline, was now mentioned by one professional in the end-line.

The approaches used to overcome the disruptions of essential health services in public health services that were mentioned by at least half of the respondents, both in the baseline and the end-line, were the allocation of telemedicine to replace face-to-face consultations; redistribution of tasks from more trained professionals to other health professionals/delegation of function; new supply chain approaches and/or dispensing of medicines through other channels; triage to identify priorities; and redirection of patients to alternative health centres.

## Discussion

Briefly, the HCPs’ accounts and perspectives on the delivery of SRH services during COVID-19 conclude that the different speeches and behaviours of the government leaders caused discredit to the health measures recommended by the WHO. There were communication problems between government bodies and the population, since at the beginning of the pandemic, it was said that people should not seek medical attention in case of having soft COVID-19 symptoms. The only way to seek assistance was if symptoms persisted and worsened. This confused the population, who was not aware on how to identify the severity of symptoms. The health systems orientation changed in the end-line interview and the population were well-orientated and learned that they should look for a doctor as soon as possible. These findings corroborate what has already been described in other studies [18–21].

In the first moment of the pandemic, services were focused almost exclusively on COVID-19 cases, and family planning was not the priority, especially in Jundiai and Santos. This same problem was observed in other diseases that the population already had or manifested during the beginning of the pandemic. Therefore, many people aggravated their already existing health problems due to lack of medical care, and other health problems arose in the interim. This is in accordance with what occurred in other settings [5, 7, 20]. With the decline of the feared cases of COVID-19 and almost normally working the health services had a very large demand for work because it was repressed.

So, the SRH services were kept in all three cities. During the pandemic, the participants needed to use the services of the PHU and they were not well cared for, there was no standard of management among professionals of the same institution, and the lack of a gynaecologist, which was already something that existed, but worsened during the pandemic. All patients and partners shown knowledge of how to get the contraceptive methods and medical appointments, it is seeking the nearest PHU. The barrier was the long-time waiting in line for appointments and exams. It was told during the COVID-19 pandemic when it got worse. In addition, many people stopped seeking medical assistance, in general, because they were afraid of being infected by COVID-19. They said that public transportation was always crowded and full of people. It was said that there are more risks of being infected in public transportation than in the hospital. Similar scenarios were also described in several other locations during the pandemic [5, 7, 22].

The quantitative component of the study showed that, at the first moment, after the pandemic was decreed, some of the services interrupted elective care for a short period. In this period, there was an organisation to face the COVID-19. Initially, there was little demand for SRH services, due to the population's fear of being contaminated. With the advent of vaccines, the fear was dissipating. When the cases of contamination and deaths by COVID-19 decreased, the demand for SRH services increased, including a demand for telemedicine as found in other places [23]. At the time of the end-line, the routine of the services was practically normalised, but there was, according to healthcare professionals and participants, a repressed demand for care. So, in the quantitative analysis, no significant changes over time were observed.

Some HCPs, due to the initial information being contradictory, increased fear of the disease and of contaminating their family members. On the end-line, it was observed that, as information about COVID-19 was standardised and vaccination started, this fear decreased. The population showed fear of seeking health services and becoming contaminated with COVID-19. The decrease in public transportation, and the consequent agglomeration generated fear in the population. These aspects changed with time and control of the pandemic, especially when the vaccines started to be used and proved to be effective and safe. However, these aspects should always be considered by health managers in possible future similar epidemics or pandemics.

### Strengths and limitations

Although developed in three affected cities from the Southeast of Brazil, using a mix of qualitative and quantitative approaches, a strength of this study is that is one of the first exploring this way the Brazilian reality of the SRH services in the pandemic era. The sample could be considered limited because it not included services and women from other regions of the country where the health crisis during the pandemic was even worse. There were healthcare professionals who did not participate in the interview due to the lack of time or because they got sick and also because the questionnaire was considered too long. An important strength is that other similar studies using a common protocol were developed in other nine countries (Burkina Faso, China, England, Ghana, Italy, Kenya, Pakistan, Thailand and United Kingdom) under the coordination of WHO, which will allow a broad picture of the disruption of SRH services around the world, comparing the results among these countries and joining the experience of each one of them.

The results of the study supported by the experience of HCPs, clients and partners, allow some recommendations to be outlined for future conditions like the world experienced between 2020–2022 with COVID-19: SHR services should be considered essential and opened in all circumstances, even in a pandemic situation; there should have doctors, especially gynaecologists and nurses, available to prescribe contraceptive methods and also exams (mammography, ultrasound, etc.); there should exist standard information and care for the population in all PHU; the service capacity should be improved throughout the advocacy, political actions and high level of communication with the population; government actions to guarantee basic needs to the population, including food, medicines and enough public transportation.

Therefore, some practical implications should be faced as lessons learned for the present and future of SRH services in the country and other places with similar resource availability. The pandemic highlighted a problem that existed in Brazil before the pandemic, which was the lack of doctors in the public health service and the difficulty in scheduling visits and exams. There is a need for alignment among health managers at different levels, municipal, state, and federal. In general, SRH is centred on women. There is a need to change this focus, facilitating through educational activities the access of men to services. In addition, contraception and STDs, in some services, were not considered a priority in the pandemic. However, participants reported they should be considered essential services in future pandemics. Finally, other lessons learned refer to the innovation that the pandemic appropriated to be developed for routine use including telemedicine to replace face-to-face visits with online consultation, task shift among several health professionals, redefinition of health priorities for the whole population, and the importance of mental health to be adequately addressed in future similar situations.

## Data Availability

The data will be available upon request as per the WHO policies. Requests for access to data can be sent to alimoa@who.int.

## Abbreviations

COC: Combined oral contraceptive
COVID-19: Coronavirus Disease 2019
HCP: Healthcare Provider/Professional
HIV: Human Immunodeficiency Virus
IDI: In-Depth Interview
IUD: Intra uterine device
FG: Focus Group
FP: Family Planning
GBV: Gender Based Violence
PHU: Primary Health Unit
PIS: Participant Information Sheet
RCS: Research Capacity Strengthening
SARA: Service Availability and Readiness Assessment
SARS-CoV-2: Severe Acute Respiratory Syndrome Coronavirus 2
SARC: Sexual Assault Referral Centre
SHAC: Sexual Health & Contraceptive Service
SRH: Sexual and Reproductive Health
STD: Sexually Transmitted Disease
STI: Sexually Transmitted Infection
UNFPA: United Nations Sexual and Reproductive Health Agency (United Nations Population Fund)
UNICAMP: State University of Campinas
VAW: Violence Against Women
WHO: World Health Organization
